# Supplementary material for: Assessing the environmental impact of medicines in Italy using data from the Italian Medicines Agency
Source: Br J Clin Pharmacol. 2025 Mar 18;91(5):1297–305. doi: 10.1002/bcp.70046 (PMC12035592; doi:10.1002/bcp.70046)
Supplement: Supplementary file 1 — SUPPORTING INFORMATION TABLE S1 Medicines excluded from lowest PNEC selection SUPPORTING INFORMATION TABLE S2 Medicines selected for the analysis SUPPORTING INFORMATION TABLE S3 Risk quotients, risk classes, PEC, PNEC and total drug utilization in kilograms for the 90 selected medicines in Italy, 2022 SUPPORTING INFORMATION TABLE S4 Risk quotients, PEC (expressed in μg/L) and total drug utilization (expressed in kg) for the 90 selected medicines in 2022 in each Italian macro‐area. PNEC are the same as reported in Table S2 and risk can be interpreted as high when ≥10, moderate when ≥1, low when ≥0.1 and insignificant otherwise. SUPPORTING INFORMATION FIGURE S1 Risk level of the 90 selected medicines in 2022 according to the Italian region [file BCP-91-1297-s002.docx]

**Supplementary material**

**Table S1** – Medicines excluded from lowest PNEC selection

| **Pharmaceutical** | **Reason** |
| --- | --- |
| Samatasvir | Still in phase II |
| Anacetrapib | Development abandoned in 2017 |
| Avasimibe | Used only in trials |
| Implitapide | Still in phase II |
| Bizelesin | Still in phase I |
| Vedroprevir | Still in phase II |
| Azelnidipine | Sold in Japan and India only |
| Triclocarban | Theoretically not used nowadays |
| Sovaprevir | Still in phase II |
| Efonidipine | Sold in Japan and India only |
| Cenicriviroc | Still in phase III |
| Linsitinib | Still in phase III |
| Rebastinib | Still in phase II |

**Table S2** – Medicines selected for the analysis

| **ATC I Class** | **ATC code** | **Substance** | **Inclusion criteria** |
| --- | --- | --- | --- |
| A | A02BC01 | omeprazole | Drug utilization |
|  | A02BC02 | pantoprazole | Drug utilization |
|  | A02BC03 | lansoprazole | Drug utilization |
|  | A02BC05 | esomeprazole | Drug utilization |
|  | A10BA02 | metformin | Drug utilization;Watch List |
|  | A11CC05 | cholecalciferol | Drug utilization |
| B | B01AC04 | clopidogrel | Drug utilization |
|  | B01AC07 | dipyridamole | Watch List |
|  | B02BX07 | lusutrombopag | PNEC |
|  | B03BA01 | cyanocobalamin | Drug utilization |
| C | C02KX01 | bosentan | PNEC |
|  | C03CA01 | furosemide | Drug utilization |
|  | C07AA05 | propranolol | Watch List |
|  | C07AB07 | bisoprolol | Drug utilization |
|  | C07AB12 | nebivolol | Drug utilization |
|  | C08CA01 | amlodipine | Drug utilization |
|  | C08CA02 | felodipine | PNEC |
|  | C08CA03 | isradipine | PNEC |
|  | C08CA07 | nisoldipine | PNEC |
|  | C08CA09 | lacidipine | PNEC |
|  | C08CA16 | clevidipine | PNEC |
|  | C09AA05 | ramipril | Drug utilization |
|  | C09CA03 | valsartan | Drug utilization |
|  | C09CA04 | irbesartan | Watch List |
|  | C09CA08 | olmesartan | Drug utilization |
|  | C10AA01 | simvastatin | Drug utilization |
|  | C10AA05 | atorvastatin | Drug utilization |
|  | C10AA07 | rosuvastatin | Drug utilization |
|  | C10AB04 | gemfibrozil | Watch List |
|  | C10AX09 | ezetimibe | Drug utilization |
|  | C10AX12 | lomitapide | PNEC |
| D | D06AX11 | rifaximin | PNEC |
| G | G01AF02 | clotrimazole | Watch List |
|  | G03AC01 | norethisterone | Watch List |
|  | G03AC03 | levonorgestrel | PNEC;Watch List |
|  | G03CA01 | ethinylestradiol | PNEC;Watch List |
|  | G03CA03 | estradiol | PNEC;Watch List |
|  | G04CA02 | tamsulosin | Drug utilization |
| H | H03AA01 | levothyroxine | Drug utilization |
| J | J01CA04 | amoxicillin | Watch List |
|  | J01DB01 | cefalexin | Watch List |
|  | J01EA01 | trimethoprim | Watch List |
|  | J01EC01 | sulfamethoxazole | Watch List |
|  | J01FA01 | erythromycin | Watch List |
|  | J01FA09 | clarithromycin | Watch List |
|  | J01FA10 | azithromycin | Watch List |
|  | J01FF01 | clindamycin | Watch List |
|  | J01MA01 | ofloxacin | Watch List |
|  | J01MA02 | ciprofloxacin | Watch List |
|  | J02AB01 | miconazole | Watch List |
|  | J02AC01 | fluconazole | Watch List |
|  | J04AK05 | bedaquiline | PNEC |
|  | J05AG05 | rilpivirine | PNEC |
|  | J05AP10 | elbasvir | PNEC |
|  | J05AP11 | grazoprevir | PNEC |
| L | L01AA01 | cyclophosphamide | Watch List |
|  | L01BC02 | fluorouracil | Watch List |
|  | L01CA05 | vinflunine | PNEC |
|  | L01DB01 | doxorubicin | Watch List |
|  | L01DB02 | daunorubicin | Watch List |
|  | L01EA01 | imatinib | PNEC |
|  | L01EA03 | nilotinib | PNEC |
|  | L01EC02 | dabrafenib | PNEC |
|  | L01ED02 | ceritinib | PNEC |
|  | L01EH01 | lapatinib | PNEC |
|  | L01EJ02 | fedratinib | PNEC |
|  | L01EX10 | midostaurin | PNEC |
|  | L02BA02 | toremifene | PNEC |
|  | L04AA06 | mycophenolate | Watch List |
| M | M01AB05 | diclofenac | Drug utilization;Watch List |
|  | M01AB14 | proglumetacin | PNEC |
|  | M01AE01 | ibuprofen | Drug utilization |
|  | M01AE03 | ketoprofen | Drug utilization |
|  | M04AA01 | allopurinol | Drug utilization;Watch List |
| N | N01AH01 | fentanyl | Watch List |
|  | N02BA01 | acetylsalicylic acid | Drug utilization |
|  | N02BE01 | paracetamol | Drug utilization |
|  | N02BF01 | gabapentin | Watch List |
|  | N05CD06 | lormetazepam | Drug utilization |
|  | N05CD08 | midazolam | Watch List |
|  | N06AB06 | sertraline | Drug utilization |
|  | N06AX16 | venlafaxine | Watch List |
| P | P01AX06 | atovaquone | PNEC |
|  | P01BF05 | piperaquine | Watch List |
|  | P02CA01 | mebendazole | Watch List |
|  | P03AC04 | permethrin | PNEC |
| R | R01AA08 | naphazoline | Drug utilization |
|  | R03DC03 | montelukast | PNEC |
|  | R06AX22 | ebastine | PNEC |
| S | S01LA01 | verteporfin | PNEC |

**Table S3** – Risk quotients, risk classes, PEC, PNEC, and total drug utilization in kilograms for the 90 selected medicines in Italy, 2022

| **Substance** | **DU (kg)** | **PEC** | **PNEC (µg/L)** | **RQ** | **Risk** |
| --- | --- | --- | --- | --- | --- |
| acetylsalicylic acid | 227292.233 | 5.285 | 18.000 | 0.294 | Low |
| allopurinol | 93013.978 | 2.163 | 20.558 | 0.105 | Low |
| amlodipine | 6819.880 | 0.156 | 0.230 | 0.689 | Low |
| amoxicillin | 300471.054 | 6.986 | 0.078 | 89.566 | High |
| atorvastatin | 24272.278 | 0.564 | 8.500 | 0.066 | Insignificant |
| atovaquone | 466.674 | 0.011 | 0.001 | 13.073 | High |
| azithromycin | 16654.818 | 0.387 | 0.019 | 20.381 | High |
| bedaquiline | 1.485 | 0.000 | 0.003 | 0.013 | Insignificant |
| bisoprolol | 3091.188 | 0.072 | 92.000 | 0.001 | Insignificant |
| bosentan | 204.196 | 0.005 | 0.003 | 1.752 | Moderate |
| cefalexin | 2026.775 | 0.047 | 0.080 | 0.589 | Low |
| ceritinib | 2.403 | 0.000 | 0.001 | 0.049 | Insignificant |
| cholecalciferol | 205.671 | 0.005 | 0.014 | 0.347 | Low |
| ciprofloxacin | 21755.377 | 0.506 | 0.089 | 5.683 | Moderate |
| clarithromycin | 23978.310 | 0.558 | 0.120 | 4.646 | Moderate |
| clevidipine | 0.141 | 0.000 | 0.001 | 0.003 | Insignificant |
| clindamycin | 10072.303 | 0.234 | 0.044 | 5.322 | Moderate |
| clopidogrel | 19509.894 | 0.454 | 3.214 | 0.141 | Low |
| clotrimazole | 4396.261 | 0.102 | 0.020 | 5.111 | Moderate |
| cyanocobalamin | 42.090 | 0.001 | 33608.828 | 0.000 | Insignificant |
| cyclophosphamide | 176.731 | 0.004 | 6.964 | 0.001 | Insignificant |
| dabrafenib | 195.258 | 0.005 | 0.001 | 3.815 | Moderate |
| daunorubicin | 0.460 | 0.000 | 0.218 | 0.000 | Insignificant |
| diclofenac | 34473.326 | 0.802 | 0.050 | 16.031 | High |
| dipyridamole | 38.734 | 0.001 | 0.005 | 0.169 | Low |
| doxorubicin | 5.982 | 0.000 | 0.170 | 0.001 | Insignificant |
| ebastine | 726.463 | 0.017 | 0.003 | 6.054 | Moderate |
| elbasvir | 1.319 | 0.000 | 0.001 | 0.050 | Insignificant |
| erythromycin | 2496.319 | 0.058 | 0.200 | 0.290 | Low |
| esomeprazole | 12250.152 | 0.285 | 0.030 | 9.500 | Moderate |
| estradiol | 214.790 | 0.005 | 0.000 | 12.485 | High |
| ethinylestradiol | 10.570 | 0.000 | 0.000 | 7.022 | Moderate |
| ezetimibe | 3556.030 | 0.083 | 0.163 | 0.507 | Low |
| fedratinib | 7.860 | 0.000 | 0.002 | 0.101 | Low |
| felodipine | 96.974 | 0.002 | 0.002 | 1.436 | Moderate |
| fentanyl | 19.350 | 0.000 | 0.171 | 0.003 | Insignificant |
| fluconazole | 1809.268 | 0.042 | 0.250 | 0.168 | Low |
| fluorouracil | 704.824 | 0.016 | 58.500 | 0.000 | Insignificant |
| furosemide | 26755.784 | 0.622 | 0.707 | 0.880 | Low |
| gabapentin | 25708.471 | 0.598 | 10.000 | 0.060 | Insignificant |
| gemfibrozil | 3107.124 | 0.072 | 0.500 | 0.144 | Low |
| grazoprevir | 2.638 | 0.000 | 0.001 | 0.065 | Insignificant |
| ibuprofen | 384053.104 | 8.930 | 0.011 | 811.775 | High |
| imatinib | 958.006 | 0.022 | 0.003 | 8.839 | Moderate |
| irbesartan | 42789.243 | 0.995 | 700.000 | 0.001 | Insignificant |
| isradipine | 2.546 | 0.000 | 0.001 | 0.042 | Insignificant |
| ketoprofen | 53280.932 | 1.239 | 2.096 | 0.591 | Low |
| lacidipine | 254.206 | 0.006 | 0.000 | 12.062 | High |
| lansoprazole | 11143.394 | 0.259 | 0.192 | 1.349 | Moderate |
| lapatinib | 80.927 | 0.002 | 0.001 | 1.542 | Moderate |
| levonorgestrel | 5.615 | 0.000 | 0.000 | 8.160 | Moderate |
| levothyroxine | 102.583 | 0.002 | 12.000 | 0.000 | Insignificant |
| lomitapide | 0.346 | 0.000 | 0.001 | 0.006 | Insignificant |
| lormetazepam | 340.753 | 0.008 | 0.166 | 0.048 | Insignificant |
| lusutrombopag | 0.004 | 0.000 | 0.002 | 0.000 | Insignificant |
| mebendazole | 692.648 | 0.016 | 0.088 | 0.183 | Low |
| metformin | 1404001.809 | 32.644 | 10.000 | 3.264 | Moderate |
| miconazole | 9116.543 | 0.212 | 0.025 | 8.405 | Moderate |
| midazolam | 61.901 | 0.001 | 0.115 | 0.013 | Insignificant |
| midostaurin | 3.191 | 0.000 | 0.001 | 0.096 | Insignificant |
| montelukast | 466.593 | 0.011 | 0.002 | 4.909 | Moderate |
| mycophenolate | 15605.201 | 0.363 | 0.132 | 2.749 | Moderate |
| naphazoline | 126.893 | 0.003 | 0.370 | 0.008 | Insignificant |
| nebivolol | 1896.279 | 0.044 | 1.838 | 0.024 | Insignificant |
| nilotinib | 303.610 | 0.007 | 0.001 | 4.770 | Moderate |
| nisoldipine | 0.000 | 0.000 | 0.002 | 0.000 | Insignificant |
| norethisterone | 53.729 | 0.001 | 0.001 | 2.498 | Moderate |
| ofloxacin | 23554.914 | 0.548 | 0.026 | 21.064 | High |
| olmesartan | 20167.312 | 0.469 | 0.004 | 116.643 | High |
| omeprazole | 9615.485 | 0.224 | 0.280 | 0.798 | Low |
| pantoprazole | 31952.477 | 0.743 | 0.681 | 1.091 | Moderate |
| paracetamol | 986193.786 | 22.930 | 134.000 | 0.171 | Low |
| permethrin | 1728.905 | 0.040 | 0.000 | 200.992 | High |
| piperaquine | 3.318 | 0.000 | 0.001 | 0.073 | Insignificant |
| proglumetacin | 4.932 | 0.000 | 0.002 | 0.067 | Insignificant |
| propranolol | 2662.680 | 0.062 | 0.100 | 0.619 | Low |
| ramipril | 5193.455 | 0.121 | 0.110 | 1.102 | Moderate |
| rifaximin | 30199.349 | 0.702 | 0.002 | 283.128 | High |
| rilpivirine | 160.280 | 0.004 | 0.003 | 1.467 | Moderate |
| rosuvastatin | 7417.417 | 0.172 | 0.269 | 0.640 | Low |
| sertraline | 11597.071 | 0.270 | 0.009 | 28.685 | High |
| simvastatin | 10659.184 | 0.248 | 2.630 | 0.094 | Insignificant |
| sulfamethoxazole | 3662.083 | 0.085 | 0.100 | 0.851 | Low |
| tamsulosin | 104.986 | 0.002 | 0.346 | 0.007 | Insignificant |
| toremifene | 1.503 | 0.000 | 0.000 | 0.116 | Low |
| trimethoprim | 17064.212 | 0.397 | 0.500 | 0.794 | Low |
| valsartan | 44002.931 | 1.023 | 560.000 | 0.002 | Insignificant |
| venlafaxine | 8961.491 | 0.208 | 0.006 | 34.158 | High |
| verteporfin | 0.013 | 0.000 | 0.002 | 0.000 | Insignificant |
| vinflunine | 0.170 | 0.000 | 0.001 | 0.004 | Insignificant |

**Table S4** - Risk quotients, PEC (expressed in µg/L), and total drug utilization (expressed in kgs) for the 90 selected medicines in 2022 in each Italian macro-area. PNEC are the same as reported in Table S2, and risk can be interpreted as high when ≥10, moderate when ≥1, low when ≥0.1, insignificant otherwise.

| **Substance** | **DU North** | **DU Centre** | **DU South** | **PEC North** | **PEC Centre** | **PEC South** | **RQ North** | **RQCentre** | **RQ South** |
| --- | --- | --- | --- | --- | --- | --- | --- | --- | --- |
| acetylsalicylic acid | 107474.727 | 44373.528 | 75443.978 | 5.377 | 5.188 | 5.214 | 0.299 | 0.288 | 0.29 |
| allopurinol | 21114.803 | 39746.685 | 32152.49 | 1.056 | 4.647 | 2.222 | 0.051 | 0.226 | 0.108 |
| amlodipine | 3163.211 | 1466.741 | 2189.928 | 0.158 | 0.171 | 0.151 | 0.688 | 0.746 | 0.658 |
| amoxicillin | 61041.16 | 136030.433 | 103399.461 | 3.054 | 15.904 | 7.146 | 39.154 | 203.892 | 91.621 |
| atorvastatin | 11075.473 | 4617.659 | 8579.146 | 0.554 | 0.54 | 0.593 | 0.065 | 0.064 | 0.07 |
| atovaquone | 81.805 | 329.602 | 55.267 | 0.004 | 0.039 | 0.004 | 4.931 | 46.427 | 4.602 |
| azithromycin | 3520.36 | 6605.954 | 6528.503 | 0.176 | 0.772 | 0.451 | 9.27 | 40.648 | 23.748 |
| bedaquiline | 0.47 | 0.827 | 0.188 | 0 | 0 | 0 | 0.009 | 0.035 | 0.005 |
| bisoprolol | 586.458 | 1471.737 | 1032.993 | 0.029 | 0.172 | 0.071 | 0 | 0.002 | 0.001 |
| bosentan | 43.51 | 80.033 | 80.653 | 0.002 | 0.009 | 0.006 | 0.803 | 3.453 | 2.057 |
| cefalexin | 498.261 | 930.899 | 597.615 | 0.025 | 0.109 | 0.041 | 0.312 | 1.36 | 0.516 |
| ceritinib | 0.986 | 0.878 | 0.54 | 0 | 0 | 0 | 0.043 | 0.089 | 0.032 |
| cholecalciferol | 120.305 | 39.541 | 45.825 | 0.006 | 0.005 | 0.003 | 0.437 | 0.336 | 0.230 |
| ciprofloxacin | 4791.997 | 7790.211 | 9173.168 | 0.24 | 0.911 | 0.634 | 2.694 | 10.233 | 7.124 |
| clarithromycin | 4987.696 | 7865.095 | 11125.52 | 0.25 | 0.92 | 0.769 | 2.08 | 7.663 | 6.408 |
| clevidipine | 0.046 | 0.056 | 0.039 | 0 | 0 | 0 | 0.002 | 0.006 | 0.003 |
| clindamycin | 2059.145 | 4973.32 | 3039.839 | 0.103 | 0.581 | 0.21 | 2.341 | 13.215 | 4.775 |
| clopidogrel | 4956.125 | 7614.789 | 6938.98 | 0.248 | 0.89 | 0.48 | 0.077 | 0.277 | 0.149 |
| clotrimazole | 979.85 | 2041.408 | 1375.003 | 0.049 | 0.239 | 0.095 | 2.451 | 11.933 | 4.752 |
| cyanocobalamin | 19.55 | 8.729 | 13.811 | 0.001 | 0.001 | 0.001 | 0 | 0 | 0 |
| cyclophosphamide | 41.756 | 84.711 | 50.264 | 0.002 | 0.01 | 0.003 | 0 | 0.001 | 0 |
| dabrafenib | 40.038 | 98.28 | 56.94 | 0.002 | 0.011 | 0.004 | 1.683 | 9.656 | 3.307 |
| daunorubicin | 0.1 | 0.204 | 0.156 | 0 | 0 | 0 | 0 | 0 | 0 |
| diclofenac | 15482.158 | 6724.279 | 12266.889 | 0.775 | 0.786 | 0.848 | 15.492 | 15.723 | 15.957 |
| dipyridamole | 9.335 | 19.902 | 9.497 | 0 | 0.002 | 0.001 | 0.087 | 0.436 | 0.123 |
| doxorubicin | 1.116 | 2.969 | 1.897 | 0 | 0 | 0 | 0 | 0.002 | 0.001 |
| ebastine | 133.944 | 314.322 | 278.198 | 0.007 | 0.037 | 0.019 | 2.402 | 13.171 | 6.892 |
| elbasvir | 0.143 | 0.521 | 0.655 | 0 | 0 | 0 | 0.012 | 0.099 | 0.073 |
| erythromycin | 587.104 | 1032.674 | 876.54 | 0.029 | 0.121 | 0.061 | 0.147 | 0.604 | 0.303 |
| esomeprazole | 1952.242 | 5848.504 | 4449.407 | 0.098 | 0.684 | 0.308 | 3.258 | 22.806 | 10.257 |
| estradiol | 339.660 | 106.464 | 132.344 | 0.017 | 0.012 | 0.009 | 42.485 | 31.117 | 22.867 |
| ethinylestradiol | 6.125 | 1.927 | 2.602 | 0 | 0 | 0 | 8.756 | 6.436 | 5.139 |
| ezetimibe | 1611.726 | 748.697 | 1195.607 | 0.081 | 0.088 | 0.083 | 0.495 | 0.537 | 0.507 |
| fedratinib | 0.648 | 4.452 | 2.76 | 0 | 0.001 | 0 | 0.018 | 0.286 | 0.105 |
| felodipine | 18.03 | 57.616 | 21.328 | 0.001 | 0.007 | 0.001 | 0.575 | 4.29 | 0.939 |
| fentanyl | 11.126 | 3.129 | 5.096 | 0.001 | 0.000 | 0.000 | 0.003 | 0.002 | 0.002 |
| fluconazole | 337.143 | 682.294 | 789.831 | 0.017 | 0.08 | 0.055 | 0.067 | 0.319 | 0.218 |
| fluorouracil | 174.182 | 269.215 | 261.426 | 0.009 | 0.031 | 0.018 | 0 | 0.001 | 0 |
| furosemide | 5720 | 12608.467 | 8427.317 | 0.286 | 1.474 | 0.582 | 0.405 | 2.086 | 0.824 |
| gabapentin | 8193.999 | 11892.598 | 5621.874 | 0.41 | 1.39 | 0.389 | 0.041 | 0.139 | 0.039 |
| gemfibrozil | 799.308 | 1423.278 | 884.538 | 0.04 | 0.166 | 0.061 | 0.08 | 0.333 | 0.122 |
| grazoprevir | 0.286 | 1.042 | 1.31 | 0 | 0 | 0 | 0.015 | 0.129 | 0.096 |
| ibuprofen | 185815.682 | 81193.76 | 117043.661 | 9.297 | 9.493 | 8.089 | 845.157 | 862.956 | 735.407 |
| imatinib | 205.774 | 455.82 | 296.412 | 0.01 | 0.053 | 0.02 | 4.085 | 21.147 | 8.13 |
| irbesartan | 10123.216 | 16597.955 | 16068.072 | 0.506 | 1.94 | 1.111 | 0.001 | 0.003 | 0.002 |
| isradipine | 0.439 | 0.977 | 1.13 | 0 | 0 | 0 | 0.016 | 0.082 | 0.056 |
| ketoprofen | 23736.918 | 10429.351 | 19114.663 | 1.188 | 1.219 | 1.321 | 0.567 | 0.582 | 0.630 |
| lacidipine | 55.502 | 115.655 | 83.049 | 0.003 | 0.014 | 0.006 | 5.667 | 27.595 | 11.714 |
| lansoprazole | 2059.518 | 5467.012 | 3616.864 | 0.103 | 0.639 | 0.25 | 0.537 | 3.329 | 1.302 |
| lapatinib | 13.748 | 28.217 | 38.962 | 0.001 | 0.003 | 0.003 | 0.564 | 2.704 | 2.207 |
| levonorgestrel | 3.277 | 1.141 | 0.926 | 0 | 0 | 0 | 10.249 | 8.334 | 3.999 |
| levothyroxine | 24.486 | 44.506 | 33.591 | 0.001 | 0.005 | 0.002 | 0 | 0 | 0 |
| lomitapide | 0.133 | 0.066 | 0.146 | 0 | 0 | 0 | 0.005 | 0.006 | 0.007 |
| lormetazepam | 62.938 | 213.777 | 64.038 | 0.003 | 0.025 | 0.004 | 0.019 | 0.151 | 0.027 |
| lusutrombopag | 0.001 | 0.002 | 0.001 | 0 | 0 | 0 | 0 | 0 | 0 |
| mebendazole | 160.331 | 328.987 | 203.33 | 0.008 | 0.038 | 0.014 | 0.091 | 0.437 | 0.16 |
| metformin | 284370.474 | 588134.109 | 531497.227 | 14.228 | 68.76 | 36.734 | 1.423 | 6.876 | 3.673 |
| miconazole | 1597.412 | 3841.645 | 3677.486 | 0.08 | 0.449 | 0.254 | 3.169 | 17.809 | 10.078 |
| midazolam | 12.448 | 27.496 | 21.957 | 0.001 | 0.003 | 0.002 | 0.005 | 0.028 | 0.013 |
| midostaurin | 0.67 | 1.635 | 0.885 | 0 | 0 | 0 | 0.043 | 0.247 | 0.079 |
| montelukast | 93.002 | 192.262 | 181.328 | 0.005 | 0.022 | 0.013 | 2.105 | 10.171 | 5.671 |
| mycophenolate | 2861.163 | 7795.064 | 4948.974 | 0.143 | 0.911 | 0.342 | 1.084 | 6.904 | 2.591 |
| naphazoline | 27.852 | 59.653 | 39.388 | 0.001 | 0.007 | 0.003 | 0.004 | 0.019 | 0.007 |
| nebivolol | 379.693 | 800.234 | 716.353 | 0.019 | 0.094 | 0.05 | 0.01 | 0.051 | 0.027 |
| nilotinib | 68.128 | 98.994 | 136.487 | 0.003 | 0.012 | 0.009 | 2.303 | 7.82 | 6.374 |
| nisoldipine |  | 0 | 0 |  | 0 | 0 |  | 0 | 0 |
| norethisterone | 21.734 | 12.249 | 19.746 | 0.001 | 0.001 | 0.001 | 2.175 | 2.864 | 2.73 |
| ofloxacin | 4962.679 | 9451.613 | 9140.622 | 0.248 | 1.105 | 0.632 | 9.55 | 42.5 | 24.298 |
| olmesartan | 7850.927 | 3569.703 | 8746.683 | 0.393 | 0.417 | 0.605 | 97.711 | 103.816 | 150.38 |
| omeprazole | 1532.085 | 3625.139 | 4458.261 | 0.077 | 0.424 | 0.308 | 0.274 | 1.514 | 1.1 |
| pantoprazole | 6914.531 | 13928.816 | 11109.131 | 0.346 | 1.628 | 0.768 | 0.508 | 2.391 | 1.127 |
| paracetamol | 170395.65 | 521775.659 | 294022.477 | 8.525 | 61.002 | 20.321 | 0.064 | 0.455 | 0.152 |
| permethrin | 457.486 | 647.233 | 624.186 | 0.023 | 0.076 | 0.043 | 114.445 | 378.346 | 215.703 |
| piperaquine | 0.439 | 2.221 | 0.658 | 0 | 0 | 0 | 0.021 | 0.245 | 0.043 |
| proglumetacin | 0.288 | 0.6 | 4.044 | 0 | 0 | 0 | 0.008 | 0.041 | 0.163 |
| propranolol | 446.849 | 1476.887 | 738.945 | 0.022 | 0.173 | 0.051 | 0.224 | 1.727 | 0.511 |
| ramipril | 2616.369 | 1111.554 | 1465.531 | 0.131 | 0.13 | 0.101 | 1.194 | 1.186 | 0.924 |
| rifaximin | 6523.591 | 12207.386 | 11468.372 | 0.326 | 1.427 | 0.793 | 131.608 | 575.479 | 319.612 |
| rilpivirine | 34.358 | 101.796 | 24.127 | 0.002 | 0.012 | 0.002 | 0.677 | 4.685 | 0.657 |
| rosuvastatin | 3652.356 | 1405.255 | 2359.806 | 0.183 | 0.164 | 0.163 | 0.678 | 0.61 | 0.605 |
| sertraline | 2461.388 | 6594.448 | 2541.236 | 0.123 | 0.771 | 0.176 | 13.101 | 82.018 | 18.685 |
| simvastatin | 4840.597 | 2161.669 | 3656.918 | 0.242 | 0.253 | 0.253 | 0.092 | 0.096 | 0.096 |
| sulfamethoxazole | 1708.905 | 740.764 | 1212.414 | 0.085 | 0.087 | 0.084 | 0.855 | 0.866 | 0.838 |
| tamsulosin | 21.509 | 53.178 | 30.299 | 0.001 | 0.006 | 0.002 | 0.003 | 0.018 | 0.006 |
| toremifene | 0.437 | 0.74 | 0.326 | 0 | 0 | 0 | 0.073 | 0.288 | 0.075 |
| trimethoprim | 8026.04 | 3440.008 | 5598.163 | 0.402 | 0.402 | 0.387 | 0.803 | 0.804 | 0.774 |
| valsartan | 18919.947 | 8692.322 | 14440.684 | 0.947 | 1.016 | 0.998 | 0.002 | 0.002 | 0.002 |
| venlafaxine | 1882.582 | 4737.363 | 2341.547 | 0.094 | 0.554 | 0.162 | 15.441 | 90.796 | 26.53 |
| verteporfin | 0.002 | 0.008 | 0.002 | 0 | 0 | 0 | 0 | 0 | 0 |
| vinflunine | 0.027 | 0.104 | 0.04 | 0 | 0 | 0 | 0.001 | 0.012 | 0.003 |


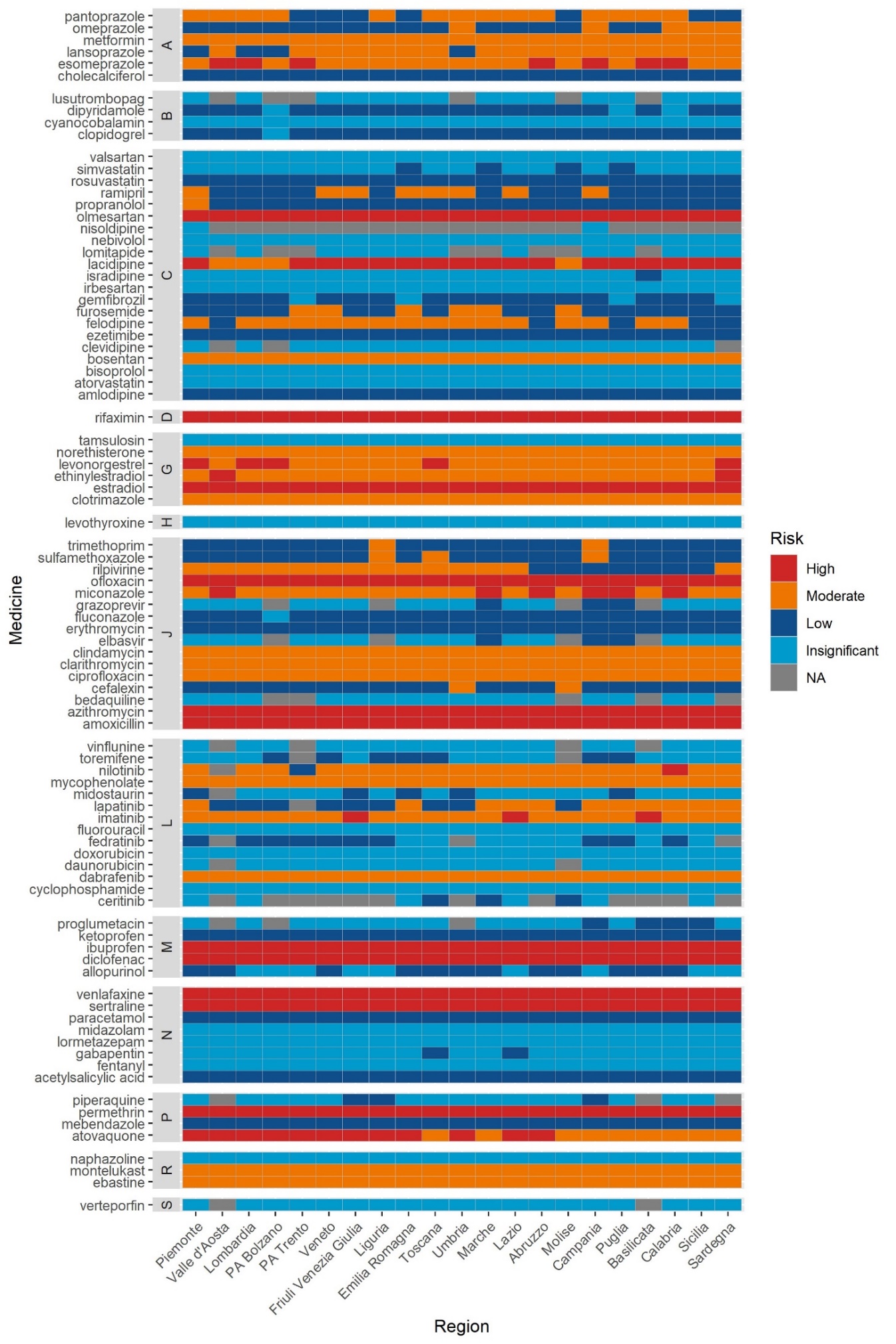


**Figure S1** – Risk level of the 90 selected medicines in 2022 according to the Italian region
